# Supplementary figures and images for: Association of HSF1 Genetic Variation with Heat Tolerance in Chinese Cattle
Source: Animals (Basel). 2019 Nov 25;9(12):1027. doi: 10.3390/ani9121027 (PMC6941060; doi:10.3390/ani9121027)

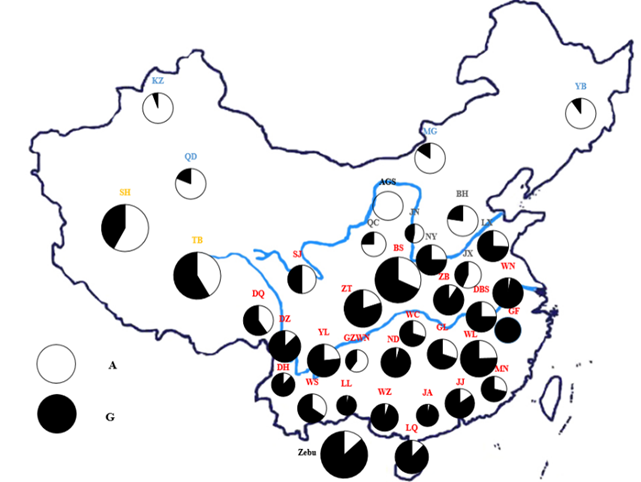

Supplement: Supplementary file 1 [file animals-09-01027-s001.zip › Supplementary File/Figure 1. Geographical distribution of two variants among 35 Chinese breeds as well as Augus and zebu population..tif]
